# Constant Voltage Iontophoresis Technique to Deliver Terbinafine via Transungual Delivery System: Formulation Optimization Using Box–Behnken Design and In Vitro Evaluation

**DOI:** 10.3390/pharmaceutics13101692

**Published:** 2021-10-15

**Authors:** Anroop B. Nair, Bandar E. Al-Dhubiab, Jigar Shah, Bapi Gorain, Shery Jacob, Mahesh Attimarad, Nagaraja Sreeharsha, Katharigatta N. Venugopala, Mohamed A. Morsy

**Affiliations:** 1 Department of Pharmaceutical Sciences, College of Clinical Pharmacy, King Faisal University, Al-Ahsa 31982, Saudi Arabia; baldhubiab@kfu.edu.sa (B.E.A.-D.); mattimarad@kfu.edu.sa (M.A.); sharsha@kfu.edu.sa (N.S.); kvenugopala@kfu.edu.sa (K.N.V.); momorsy@kfu.edu.sa (M.A.M.); 2 Department of Pharmaceutics, Institute of Pharmacy, Nirma University, Ahmedabad 382481, India; jigsh12@gmail.com; 3 School of Pharmacy, Faculty of Health and Medical Sciences, Taylor’s University, Subang Jaya 47500, Malaysia; bapi.gn@gmail.com; 4 Centre for Drug Delivery and Molecular Pharmacology, Faculty of Health and Medical Sciences, Taylor’s University, Subang Jaya 47500, Malaysia; 5 Department of Pharmaceutical Sciences, College of Pharmacy, Gulf Medical University, Ajman 4184, United Arab Emirates; sheryjacob6876@gmail.com; 6 Department of Pharmaceutics, Vidya Siri College of Pharmacy, Off Sarjapura Road, Bangalore 560035, India; 7 Department of Biotechnology and Food Technology, Durban University of Technology, Durban 4000, South Africa; 8 Department of Pharmacology, Faculty of Medicine, Minia University, El-Minia 61511, Egypt

**Keywords:** nail permeation, onychomycosis, enhancer, low voltage iontophoresis, Box–Behnken model, antifungal study

## Abstract

Topical therapy of antifungals is primarily restricted due to the low innate transport of drugs through the thick multi-layered keratinized nail plate. The objective of this investigation was to develop a gel formulation, and to optimize and evaluate the transungual delivery of terbinafine using the constant voltage iontophoresis technique. Statistical analysis was performed using Box–Behnken design to optimize the transungual delivery of terbinafine by examining crucial variables namely concentration of polyethylene glycol, voltage, and duration of application (2–6 h). Optimization data in batches (F1–F17) demonstrated that chemical enhancer, applied voltage, and application time have influenced terbinafine nail delivery. Higher ex vivo permeation and drug accumulation into the nail tissue were noticed in the optimized batch (F8) when compared with other batches (F1–F17). A greater amount of terbinafine was released across the nails when the drug was accumulated by iontophoresis than the passive counterpart. A remarkably higher zone of inhibition was observed in nails with greater drug accumulation due to iontophoresis, as compared to the passive process. The results here demonstrate that the optimized formulation with low voltage iontophoresis could be a viable and alternative tool in the transungual delivery of terbinafine, which in turn could improve the success rate of topical nail therapy in onychomycosis.

## 1. Introduction

Nail diseases are one of the most frequently occurred topical disorders affecting nearly 6% of the entire human population [1]. The leading causes of nail diseases are fungal infections, psoriasis, and paronychia. Onychomycosis, also termed as *Tinea unguium*, is a contagious fungal disease involving the nail unit that causes thickening, discoloration, hardening, fracture, and detachment of the nail plate from the nail bed, and paronychia [2] besides affecting the underneath tissue of fingers and toes [3]. The main contributing factors affecting this disease are the existence of athlete’s foot, cancer, immunodeficiency, psoriasis, diabetes, and older age [4,5]. Dermatophytes (*Trichophyton*, *Microsporum* or *Epidermophyton genera*) are the most prevalent causative organism for onychomycosis, whereas non-dermatophytic molds (*Scopulariopsis*, *Fusarium* spp., *Scytalidium*, *Acremonium*, and *Aspergillus* spp.) and yeasts (*Candida albicans*, *C. parapsilosis*) are also the sources of this infection [6]. Moreover, toenails are highly susceptible to this infective disorder when compared to fingernails, which demands chronic treatment [7]. This chronic fungal nail infection generally makes the patient discomfort, isolate from social activities, and negatively impacts the quality of life [8].

Currently, oral and topical therapy are utilized either individually or in combination for the management of onychomycosis and recalcitrant dermatophytosis [7,9]. Oral therapy primarily includes terbinafine, itraconazole, and fluconazole, in addition to the new antifungals like fosravuconazole being tested [2]. Nevertheless, the clinical and mycological success rate with oral antifungal drugs for long-term therapy is reported to be moderate, with terbinafine showing more efficacy than azoles [10]. The major limitation in oral administration includes chronic therapy because of the low drug level at the target site owing to the poor perfusion in the nail bed. In most cases, the oral administration of antimycotic agents demonstrate low efficacy with fewer patients attaining clinical cure and most experiencing relapses once the treatment is stopped [7]. In addition, these drugs can induce hepatotoxicity, gastrointestinal tract irritation, drug–drug interactions, increased microbial resistance, and systemic adverse effects [11,12]. On the other hand, topical therapy is a relatively safe and preferred route of administration since it possesses several advantages such as greater vicinity to the site, provides target drug delivery, improved patient compliance, and is exempted from systemic adverse effects [13,14]. Hence, there is a growing demand for developing new delivery systems, which are non-invasive, effective, non-toxic, and have increased patient convenience than traditional oral therapy. However, the cure rate of topical monotherapy with antifungal formulations of efinaconazole, tavaborole, ciclopirox, and amorolfine is relatively low (<20%) [2,15,16]. It has been realized that the most effective way of treating onychomycosis is to deliver a potent antifungal agent at a concentration above the minimum inhibitory concentration of the infected pathogen at the targeted site for the desired period [17]. At present, various commercial antifungal dosage forms such as nail lacquers, creams, ointments, gels, and solutions are available for topical application [18]. In addition, vesicular formulations like liposomes, ethosomes, transfersomes, nanoemulsions, and microneedles were investigated for transungual drug delivery [19,20]. Synthetic chemists have also attempted to improve nail permeation by modifying the structure of existing therapeutic molecules [21,22]. However, the delivery of antimycotic agents is limited in topical therapy by the fully keratinized nail plate, which is a thick multi-layered complex structure consisting of dead keratin cells and is strongly connected by intercellular links [23]. Since oral and topical therapy has certain constraints, investigation on new pharmacotherapy based on ungual and transungual drug delivery systems has gained much attention [3,19,24].

To augment the trans-nail transport of actives, different classes of chemical substances like thiols/mercaptans, keratolytics, inorganic salts, glycols, complexing species, reducing, and oxidizing agents were utilized [1,25]. They were reported to disrupt the compacted keratin fibrils by breaking their intercellular bonds to allow increased permeation of the drug into a deeper nail bed. Potent penetration enhancers that are effective in skin delivery are unlikely to be useful in nail drug delivery mainly due to the differences in their barriers and chemical composition [26]. It has also been disclosed that the drug permeation across the nail unit was extremely dictated by formulation components, physicochemical characteristics of active, amount of penetration enhancer, and the degree of nail hydration [15]. Most of the antifungal agents available are hydrophobic and possess low intrinsic transungual permeability, as the nail plate behaves as a hydrophilic gel matrix on hydration [13]. It was revealed that the application of aqueous or hydro-alcoholic formulations leads to a better cure rate due to increased nail hydration and subsequent steady drug permeation [27,28]. Thus the selection of vehicles also plays a pivotal role in the transungual drug delivery of antimycotic agents [29].

Furthermore, both mechanical and physical approaches are considered more effective than passive counterparts in delivering antifungal agents into and across the nail unit [1,15]. In general, iontophoresis, laser, microporation, ultrasound, ablation, debridement, etching, avulsion, dorsal nail abrasion, and nail trephination were attempted by researchers to enhance the transungual drug delivery [20,30]. Few studies have assessed the potential of combining both chemical and physical methods to increase the transungual permeation of antifungal agents [15,31,32]. Among these physical enhancement methods, the direct current iontophoresis seems to be extensively studied and found more effective and promising [33,34]. However, the major concern with constant current iontophoresis is the requirement of high voltage due to the higher resistance offered by the nail unit. In this context, constant voltage iontophoresis using a low current could be more tolerable to the patients and can provide effective drug delivery. Typically, in constant voltage iontophoresis, the voltage is fixed while the current varies with the change in resistance of the barrier.

Terbinafine is a synthetic allylamine group of antifungal agents reported to be effective against different kinds of dermatophytes that cause onychomycosis by inhibiting squalene monooxygenase enzyme, thereby hinder sterol synthesis in fungi [35]. Low minimum fungicidal concentration (0.003–0.006 µg/mL) [36] and the possibility of anodal iontophoresis due to positive charge [37,38] instigated this present research to optimize the transungual delivery of terbinafine for improved delivery using constant low voltage iontophoresis technique. Thus, a three-factor, three-level (3 × 3) Box–Behnken statistical design was used for optimization and delivery of terbinafine by evaluating the effect of three independent variables (concentration of polyethylene glycol, applied voltage, and duration of application) on dependent variables like terbinafine permeation (across the nail) and drug accumulation into the nail tissue. The optimized formulation was characterized and assessed for in vitro antifungal activity.

## 2. Materials and Methods

### 2.1. Materials

Terbinafine hydrochloride (Erva Healthcare, Rajkot, India), was obtained as a gift. Polyvinylpyrrolidone K 30 (PVP K30), tween 80, polyethylene glycol 200 (PEG 200), polyethylene glycol 400 (PEG 400), and propylene glycol were procured from Sigma Aldrich, St. Louis, MO, USA. Sabouraud dextrose agar (SDA) and potato dextrose agar were obtained from HiMedia, Mumbai, India. Ethanol, acetonitrile, and orthophosphoric acid were purchased from Qualikems, New Delhi, India.

### 2.2. High-Performance Liquid Chromatography (HPLC)

Quantification of terbinafine was carried out using a Prominence HPLC system (Shimadzu, Kyoto, Japan) with a UV-visible absorbance detector. Optimized chromatographic separation of terbinafine was achieved using a C18 RP-HPLC column (Zorbax, 150 mm × 4.6 mm, the internal diameter of particle size is 5 μm) using a mobile phase consisting of water and acetonitrile [60:40 and the pH adjusted to 2 with orthophosphoric acid (0.183 M, 1.6 mL)]. The elution was carried out isocratically at a flow rate of 1.0 mL/min, while the volume of sample injected was 20 µL and the observed retention time in the current chromatographic conditions was 2.42 min. HPLC instrument for the analysis was set at 20 µL and the retention time was 2.42 min. The optimized method for terbinafine was also validated according to ICH guidelines, which demonstrated good linearity (5–400 ng/mL; r^2^ = 0.9995), the limit of detection (4.07 ng/mL), the limit of quantification (12.32 ng/mL), after the system suitability test. In addition, this analytical method showed good accuracy (−1.45% and −1.94%) and precision (1.62% and 1.45% for intra and inter-day, respectively).

### 2.3. Screening of Chemical Enhancers

Nail clippings (2 mm × 2 mm) from human volunteers were weighed, soaked in 2 mL solution of the drug (10 mg/mL), and 20% *w*/*w* enhancer (citric acid, ethyl alcohol, glycerin, PEG 1000, PEG 200, PEG 3350, PEG 400, propylene glycol, salicylic acid, span 20, span 40, span 60, span 80, transcutol P, tween 20, tween 40, tween 60, tween 80, and urea) for a duration of 24 h. The nail was taken out and the surface drug was cleaned by following a standardized procedure. In brief, the nail surface was scrubbed by applying a cotton sponge (wetted in 95% ethanol) and flushed with 1 mL ethanol, and the whole process was repeated 5 times. The surface of the nail plate was wiped, the weight of each nail was recorded and dissolved in sodium hydroxide solution (1 M, 1.5 mL). The drug was separated according to the method reported previously [37]. Briefly, the dissolved solution of nails in the vials was neutralized by adding hydrochloric acid (5 M, 200 µL). The extraction of terbinafine to hexane (3 mL) was performed by manually shaking the vials for 30 min. To complete the extraction, the mixture was centrifuged at 5000 rpm for 8 min. The supernatant layer was separated and added isopropyl alcohol: sulfuric acid (15:85, 1 mL) and stirred for 30 min. The bottom layer, which comprises the larger concentration of the drug, was collected separately and analyzed by HPLC.

### 2.4. Preparation of Preliminary Batches

The composition of various batches of gels (T1–T6) prepared is presented in Table 1. Briefly, the required amount of Tween 80 (5% *w*/*w*), PEG 200/400 (15 or 30% *w*/*w*), propylene glycol (10% *w*/*w*) were dispersed in purified water by continuous mixing and heating (~45–50 °C). The solution mixture was later cool down to room temperature, ethanol (20% *w*/*w*), terbinafine HCl (4% *w*/*w*), PVP K30 (10 or 20% *w*/*w*) were added and blended in a laboratory shaker (EIE 405, EIE Instruments, Ahmedabad, India) for 12 h to obtain a clear homogeneous gel. The formulation pH was fixed at 3.8 by adding 0.1 N sodium hydroxide and the weight was adjusted with water. The prepared gel was stored in a suitable light-protected well-closed container.

#### Iontophoresis Study

As the permeation and drug accumulation was greater in batch T4 (during passive delivery), this formulation was used for evaluating the iontophoresis effect. Iontophoresis studies were carried out to assess the effect of voltage (by applying two different voltages-6 and 12 V) and application time (for 3 and 6 h) on the permeation and drug accumulation in the nail. For this, four batches (T4a, T4b, T4c, and T4d) were used as below:

Batch T4a: Applied voltage is 6V and duration of 3 h
Batch T4b: Applied voltage is 6V and duration of 6 h
Batch T4c: Applied voltage is 12V and duration of 3 h and
Batch T4d: Applied voltage is 12V and duration of 6 h.

### 2.5. Experimental Design

A Box–Behnken three-factor, three-level (3 × 3) experimental design using Design of Expert software (Version 12, Stat-Ease Inc., Suite 480, Minneapolis, MN, USA) was constructed to optimize the terbinafine formulation for transungual iontophoretic delivery. The obtained statistical evaluation helped to bring the relationship between the independent variables, i.e., PEG 400 (A), applied voltage (B), and application time at the nail surface (C) on the dependent variables, i.e., permeation of drug via the nail (R1) and drug accumulation into the nail tissue (R2). Therefore, the changes in the level of factors (+1, 0, and −1) of the independent variables (A, B, and C) were studied on the effect of dependent variables to obtain the highest drug accumulation and permeation of the drug. Software suggested seventeen batches were prepared (F1–F17) for the optimization process based on the preliminary T4 composition. The F1–F17 batches were evaluated with varying proportions of independent variables, where incorporation of experimental data of dependent variables generated polynomial equations and statistical evaluations by the software.

### 2.6. Characterization of Preliminary Formulation

#### 2.6.1. Appearance and pH

The clarity of the formulations (T1–T6) was checked by visually observing for the presence of any particles or precipitation or turbidity. Formulation pH was measured using a digital pH meter (Mettler Toledo MP-220, Greifensee, Switzerland) at room temperature.

#### 2.6.2. Drug Loading Percentage and Loading Efficacy Percentage

Accurately weighed an amount (1 g) of formulations taken in polypropylene vials, added mobile phase (5 mL), and mixed thoroughly in a shaker (EIE 405, EIE Instruments, Ahmedabad, India) until a clear homogeneous solution was obtained. The solution was further filtered through a 0.22 µm syringe filter and the filtrate was diluted suitably using mobile phase and estimated by HPLC [39]. The drug loading percentage was estimated by the equation; Drug loading percentage = (Amount of drug in the formulation)/considered formulation weight) × 100. The loading efficacy percentage was estimated by the equation; Loading efficacy percentage = (Amount of drug actually recovered/theoretical amount of drug) × 100. The drug loading percentage and loading efficacy percentage was measured six times and the results are represented as means ± SD.

#### 2.6.3. Viscosity

The viscosity of various formulations (T1–T6) was tested at room temperature (25 ± 1 °C) employing a Brookfield viscometer (LVDVI prime, Middleborough, MA, USA) [40]. The viscosity measurements were performed employing spindle S18 at an angular velocity of 30 rpm. For each formulation, the viscosity was measured six times and the results are represented as means ± SD.

### 2.7. In Vitro Permeation Studies

#### 2.7.1. Passive Permeation

Human nail clippings from healthy volunteers were used for in vitro permeation studies. Nails were hydrated by soaking in normal saline for 1 h before application and seated on a tailor-made adapter meant for nails. The entire assembly was placed in the middle of donor and receiver chambers of a regular Franz diffusion cell (Logan Instruments Ltd., Somerset, NJ, USA). The available area for the drug diffusion was 0.2 cm^2^. Prepared formulations (T1–T6; 500 μL) were individually kept in the donor compartment and allowed the drug to permeate for 6 h. The receiver chamber contains 5 mL of acidified water, whose pH was fixed to pH 3 (using 0.01 N HCl), the temperature was set at 37 °C and rotated at 100 rpm. Samples (1 mL) were withdrawn at regular intervals (2, 4, and 6 h) and replaced with fresh acidified water. The samples withdrawn were filtered using a syringe membrane filter (pore size of 0.2 µm; Millipore, Bedford, MA, USA) and readily estimated by the HPLC method developed. For each formulation, the permeation study was carried out six times and the cumulative amount of drug permeated are represented as means ± SD.

#### 2.7.2. Iontophoresis

The influence of low voltage iontophoresis on drug permeation from selected formulation (T4) was performed using Franz diffusion cells maintained in conditions the same as passive permeation studies described earlier. Silver–silver chloride electrodes (Alfa Aesar, Wardhill, MA, USA) were fastened in both chambers such that the electrode was 2 mm away from the surface of the nail plate. Tested formulation (T4) was placed in the donor chamber. A constant voltage DC power source (Rolex Scientific Engineers, Ambala, India) was employed for applying low voltage iontophoresis. Anodal iontophoresis was performed for specific time intervals by applying different voltages, which were measured using a digital multimeter (Mextech Mas830L, Mextech technologies, Mumbai, India).

### 2.8. Drug Accumulation into the Nail Tissue

Quantification of drug embedded in the nail was estimated after permeation studies following application of voltage or by the passive process. The diffusion area of the nail was demarcated, washed (standard protocol mentioned earlier), the drug was extracted from nail plates by the method described before, and analyzed by HPLC.

### 2.9. Characterization of Optimized Formulation

#### 2.9.1. Fourier Transform Infrared (FTIR)

Spectra of terbinafine, placebo, and chosen formulation (F8) were recorded using the FTIR spectrometer (6100, Jasco, Tokyo, Japan). The liquid samples were prepared by diluting with the required quantity of chloroform and the sample (0.1 mL) was injected into the spectrometer. A total of 32 scans were taken per sample at a resolution of 16 cm^−1^ and the IR spectra were developed in the range of 400–4000 cm^−1^.

#### 2.9.2. Differential Scanning Calorimetry (DSC)

The thermal behavior of terbinafine, PVP K30, and selected formulation (F8) was assessed using DSC (Hitachi DSC 7020, Tokyo, Japan). Weighted samples (5 mg) were kept in aluminum pans and secured non-hermetically, while a blank pan was taken as a standard reference during the study [41]. Thermograms were recorded at a particular heating rate (10 °C/min) under a nitrogen atmosphere with a heating range of 30–250 °C.

#### 2.9.3. Field Emission Scanning Electron Microscope

The morphology of the selected formulation (F8) was investigated with a field emission scanning electron microscope. Similarly, the surface feature of the treated nail plate (iontophoresis, 12 V for 6 h using formulation F8) and control nail (passive, 0 V for 6 h using formulation F8) nail plates were investigated. The formulation was freeze-dried at −80 °C (12 h) and used. The micrograph of the formulation and nail plates were captured on a JEOL microscope (JSM7600F, Tokyo, Japan). Images were captured at an acceleration voltage of 5 kV in low vacuum mode operation.

### 2.10. Drug Release from Nail

Nail plates were encapsulated with terbinafine using formulation (F8) by constant voltage iontophoresis (12 V for 6 h) or passive (0 V for 6 h) using Franz diffusion cells as described earlier on in vitro permeation studies. The dorsal side of the nail surface was cleaned (without detaching from the nail adapter) and was exposed to an external environment to simulate in vivo conditions. Fresh media was filled in the receiver chamber and samples (1 mL) were taken at specific intervals (every day for a week) and analyzed for the amount of terbinafine release. Samples collected at specific time points were passed through a 0.2 µm syringe membrane filter and readily quantified using the developed method by HPLC.

### 2.11. Antifungal Activity

SDA plates were prepared according to the manufacturer’s instructions. Briefly, 30 mL of sterile SDA was added in one Petri dish (90 mm × 15 mm) and kept for solidifying to yield a thickness of 6 mm. *Trichophyton mentagrophyte* (ATCC 24953) suspension was cultured on potato dextrose agar medium and incubated at 28 °C for 10 days as described previously [42]. SDA plates were inoculated by placing a sterile cotton-tipped applicator in a suspension containing spores (1.0 × 10^6^ CFU/mL) and spreading it over the plate surface and being permitted to dry for 15 min. Nail plates were embedded with terbinafine using formulation (F8) by iontophoresis (12 V for 6 h) or passive (0 V for 6 h) as described earlier on in vitro permeation studies. The nail surface was cleaned, the effective surface area was removed, and placed over the SDA plates such that the ventral surface of the nail was in contact with the agar medium. The seeded agar plates containing nails were incubated at 30 °C for 4 days and the diameter (mm) of the zone of inhibition was measured.

### 2.12. Stability

The physical and chemical stability of preparation used in the F8 batch was assessed by storing it in sealed glass vials (10 mL) and packed in a desiccator [43]. The product was stored in a stability chamber (TH 90S/G, Thermolab scientific, Mumbai, India) for 3 months at controlled room temperature (25 ± 2 °C) and relative humidity of 60 ± 5% as per the ICH guidelines [44]. The stability of the formulation was checked for non-homogeneity, lump formation, sedimentation, flowability, drug loading, pH, viscosity, and DSC.

### 2.13. Data Analysis

The values shown in tables/figures are mean ± SD. Statistical comparison to assess the effects of various treatments was carried out by *t*-test or ANOVA using GraphPad Prism software (version 6, GraphPad, San Diego, CA, USA). The variations between values yielding *p* < 0.05 were considered statistically significant.

## 3. Results and Discussion

### 3.1. Selection of Chemical Enhancers

**Figure 1:**
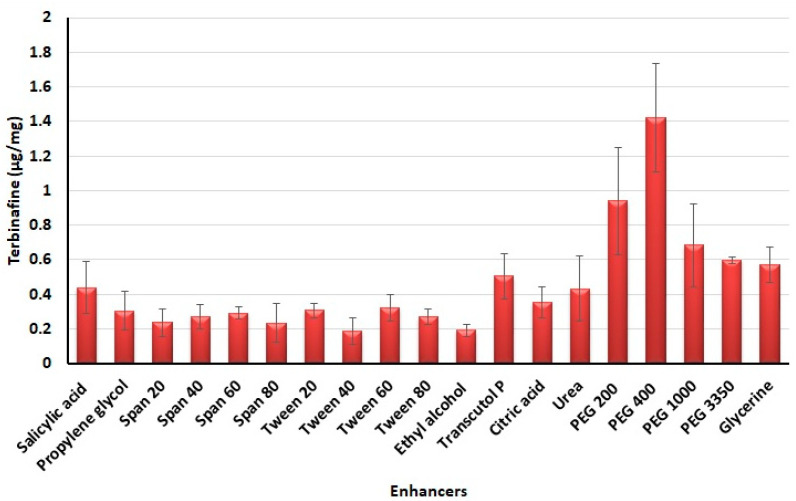
Amount of terbinafine accumulated in nail plates with various enhancers tested.

The success of topical treatment to cure nail diseases primarily depends on the potential of formulation to deliver sufficient amounts of drug into and through the nail membrane. In this context, the transport of drugs through the nail plate could be enhanced by incorporating potent chemical permeation enhancers in the formulations. Thus, identifying the most appropriate enhancers is of utmost importance to improve the drug permeation in nail plates [25]. In the current study, a high throughput approach, generally used in screening nail permeation enhancers was used to evaluate various chemicals and to identify the potent agent [45]. The most widely used ungual permeation enhancing agents were examined for their potential to enhance the terbinafine accumulation into the nail tissue and the results are presented in Figure 1. It is obvious from the figure that the drug accumulation into the nail tissue was considerably more with PEG 400 as compared to other chemicals examined in the present study. The possible mechanism for this could be due to the PEGs ability to enhance the water uptake and swelling of the nail plate that eventually becomes soft and as a result, decreases the barrier resistance and makes the nail plate more permeable [46]. In addition, the higher water uptake in the nail leads to a greater keratin hydration level, which in turn enhances the diffusion of drug molecules [46].

### 3.2. Preliminary Studies

The composition of the formulations (T1–T6) is summarized in Table 1. The prepared formulation contains various embodiments including solvent (ethanol), cosolvents (Tween 80 and propylene glycol), enhancers (PEG 200 or PEG 400), and viscosity modifier (PVP K30). Prepared batches varied for the quantity of PEGs (15 or 30% *w*/*w*) and/or PVP K30 (10 or 20% *w*/*w*) to assess their effect on various physicochemical parameters. The terbinafine level in the formulation was fixed at 4% *w*/*w*, which was the maximum amount that could be included in the prepared formulations. The results of pH, percentage drug loading, percentage laoding efficacy, viscosity, cumulative amount permeated, and drug accumulated in the nail of various formulations are shown in Table 2. No major variations in the pH, percentage drug loading and percentage laoding efficacy were noticed between various formulation batches. A minor variation in viscosity of formulations was noticed probably due to the difference in the quantity of PEGs and PVP K30. However, it appeared that the cumulative amount of terbinafine permeated (in 6 h) and accumulated in the nails varied widely between the batches. Both cumulative amount permeated (3.97 ± 1.18 µg/cm^2^) and drug accumulation (0.87 ± 0.05 µg/mg) in 6 h were superior in batch T4, and the values decreased in the order for batches; T4 > T6 > T2 > T3 > T5 > T1 (Table 2).

It is evident from the data that the formulations with PEG 400 (T4, T6, and T2) showed greater delivery (both transungual permeation and drug accumulation in the nail) in comparison to formulations containing PEG 200 (batches T3, T5, and T1). On the other hand, a minor decrease in cumulative amount permeated and drug accumulation in the nail was noticed when the PVP K30 concentration was increased from 10 to 20% (*w*/*w*) (batches; T4 versus T6 or T3 versus T5). Based on the results of drug delivery (higher permeation and drug accumulation values), batch T4 was chosen as the most suitable formulation and used in further iontophoresis studies. In the next step of the investigation, the influence of low voltage iontophoresis on terbinafine permeation and drug accumulation was evaluated. Two different voltages (6 and 12 V) at two different application time (3 and 6 h) was assessed; batch T4a (6 V and 3 h), T4b (6 V and 6 h), T4c (12 V and 3 h), and T4d (12 V and 6 h). The results indicate higher permeation (*p* < 0.0001) with batch T4d (50.22 ± 3.84 µg/cm^2^) followed by T4b (31.26 ± 2.51 µg/cm^2^), T4c (28.96 ± 4.02 µg/cm^2^) and T4a (17.42 ± 3.63 µg/cm^2^). It is evident from the data that the application time has a more prominent effect than the applied current, which signifies that the long application time could provide higher drug permeation. This improvement in drug permeation with time is due to the passive diffusion of drugs through the nail plate and this relationship has been demonstrated earlier [47,48]. Similarly, greater drug accumulation in nail (*p* < 0.005) was noticed with batch T4d (2.66 ± 0.51 µg/mg), followed by T4b (2.04 ± 0.33 µg/mg), T4c (1.73 ± 0.29 µg/mg) and T4d (1.25 ± 0.23 µg/mg). Overall, the results of preliminary trials demonstrate PEG 400, voltage, and duration have a substantial influence on the transungual delivery of terbinafine. Considering the significant effect on the permeation and drug accumulation, PEG 400, voltage, and application time was selected as independent variables. The concentration of PEG 400 (20, 30, and 40% *w*/*w*), voltage (6, 9, and 12 V), and application time (2, 4, and 6 h) were used for the optimization of design experiments.

### 3.3. Optimization Study

The use of Box–Behnken statistical design in the optimization of formulation and application parameters of pharmaceuticals has become a popular experimental design where interactions of independent and dependent variables help to obtain an optimized formulation of desired characteristics [49,50]. Using three-factor three-level optimization design, the software-derived predicted and experimental values of the 17 formulation batches (F1–F17) are summarized in Table 3. The results from the analysis were interpreted by the perturbation plot, 3D surface plot, and predicted versus actual plot. The desired criteria of permeation (45 to 55 µg/cm^2^) and drug accumulation into the nail tissue (2.65 to 3 µg/mg) were set to obtain the parameters of the independent variables for the preparation and application of the optimized formulation.

#### 3.3.1. Influence of Independent Variables on Permeation

Permeation of the drug from the formulation through the nail by application of electrical potential is an important phenomenon, which is desired for the pharmacological response of the drug in the formulation [30]. From the statistical analysis report, the model was proved to be significant with the *p*-value of <0.0001 (*p*-value < 0.05), whereas the influence of all independent variables (i.e., PEG 400, voltage, and application time) of the present design was found to be significantly (Table 4) affecting permeation of the drug.

From Table 4, it could be clearly described that the model terms A, B, C, BC, A^2^, B^2^, and C^2^ are significantly affecting the permeation of the drug. In addition, the F value of the design 911.96 denotes that the model is significant. From this analysis, it could be inferred that there will be a minimal chance (0.01%) that this large F value could occur due to noise. Furthermore, the predicted R^2^ value (0.9864) was found to be in reasonable agreement with the adjusted R^2^ value (0.9981), where the discrepancy between the predicted and adjusted R^2^ values is <0.2. In addition, the greater value of the adequate precision measure (111.361) indicated that the model represents a desirable signal-to-noise ratio, where the adequate signal could be employed to navigate the design space.

The polynomial equation in terms of the coded factor for response R1 is depicted in Equation (1).

Permeation (R1) = +32.63 + 0.9063 × A + 7.55 × B + 13.28 × C +0.5198 × AB + 0.3148 × AC + 3.59 × BC − 0.6344 × A^2^ − 1.85 × B^2^ − 1.37 × C^2^ (1)

**Figure 2:**
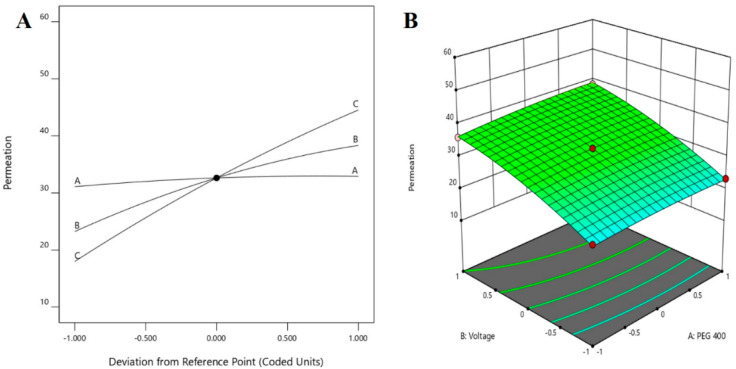
Effect of the independent variables on the permeation of drug (**A**) perturbation plot and (**B**) 3D surface plot.

**Figure 3:**
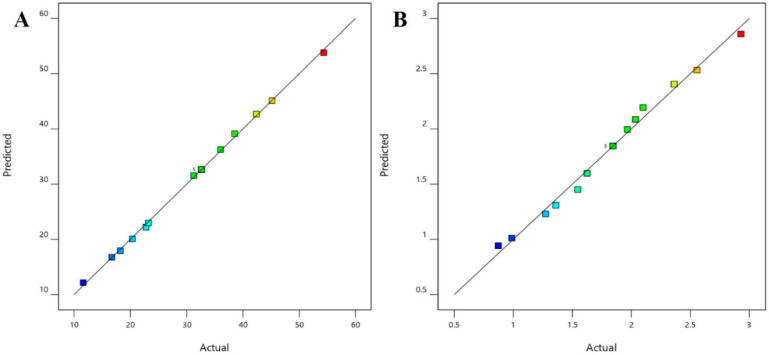
Comparison of predicted and actual values (**A**) permeation and (**B**) drug in the nail.

**Figure 4:**
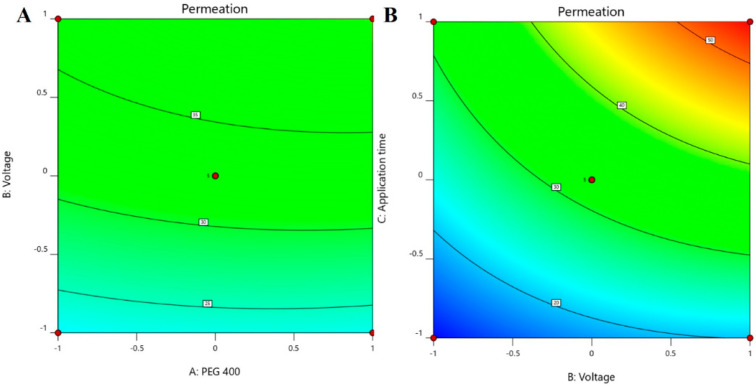
Contour plot response displaying the effect of independent variables on the permeation of drug (**A**) voltage versus PEG 400 and (**B**) application time versus voltage.

The positive coefficients of all the three model terms (i.e., A, B, and C) represent an increase of permeation with the increase in PEG 400 concentration in the formulation, and also with the application time and applied voltage. The influence of each independent variable is indicated in Table 4 where all the model terms are significantly affecting the permeation; however, the influence of model term C (i.e., application time) shows the highest when compared to the others (i.e., A and B), which is reflected by the highest coefficient value of C (13.28) when compared to the coefficient value of respective model terms in the Equation (1). At the same time, the coefficient value of model term B (7.55) is higher than the coefficient value associated with model term A (0.9063), which represents a higher effect of B (voltage) when compared to model term A (PEG 400) on nail permeation. A similar impact of the independent variables on the permeation of the drug was illustrated in the perturbation plot (Figure 2A) and the 3D surface plot (Figure 2B). It is well understood from the figures that the effect of all the three independent variables coincides with the previous explanation on the quadratic equation. In addition, the comparison of predicted and actual values of this analysis has been presented in Table 2 and Figure 3A, where the closeness of the actual and predicted values are noticeable. On the other hand, from the contour plot response, it is evident that the changes in permeation of the drug via the nail are much more promising when the applied voltage was increased than the increase in PEG content in the formulation (Figure 4A). Contrarily, the application time shows the highest impact on the permeation of the drug when compared to the applied voltage (Figure 4B). Our findings on increased permeation with the increase in PEG 400 content in the formulation is in close agreement with the existing literature [26,31,46,51], whereas an increase in voltage application during the iontophoresis process had been established for a direct relationship with permeation [52]. Moreover, the application of a constant voltage at particular nail resistance will eventually provide a constant current for the transport of drugs into and through the nail plate. Similarly, the application time of the product at the site of the application is also having a direct relationship with the permeation of the drug in the formulation [47,48].

#### 3.3.2. Effect of Independent Variables on Drug Accumulation on the Nails

Similar to permeation, the drug accumulation into the nail tissue is another important parameter for transungual therapy with antifungal formulations, wherein the drug depot in nails will eventually release and exhibit antifungal therapeutic activity [53,54]. From the obtained report by the Box–Behnken statistical design, the model can be considered significant with the *p*-value of <0.0001 (*p*-value < 0.05), whereas the influence of all the independent variables (i.e., PEG 400 content, voltage, and application time) of the experimental design is found to have significant (Table 5) effect on the drug accumulation into the nail tissue.

From Table 5, it could be demonstrated that the model expressions A, B, C, and BC are significantly (*p* < 0.05) affecting the drug accumulation into the nail tissue, where the F value of the design 87.16 suggests that the model is significant. From the analysis, similar to the permeation of the drug, it could be concluded that there will be a minimal chance (0.01%) that this large F value could arise due to noise. Furthermore, the predicted R^2^ value (0.8585) of the statistical analysis was found to be in rationale with the adjusted R^2^ value (0.9798), with a variation of <0.2. In addition, the greater value of the adequate precision measure in this model (33.404) indicated that the model exhibited a desirable signal-to-noise ratio, where the adequate signal can be employed to navigate the design space.

The polynomial equation in terms of the coded factor for response R2 is presented in Equation (2).

Accumulation into the nail tissue (R2) = +1.85 + 0.0865 × A + 0.2846 × B + 0.6738 × C + 0.0134 × AB − 0.0230 × AC + 0.1017 × BC − 0.0198 × A^2^ − 0.0158 × B^2^ − 0.0299 × C^2^ (2)

**Figure 5:**
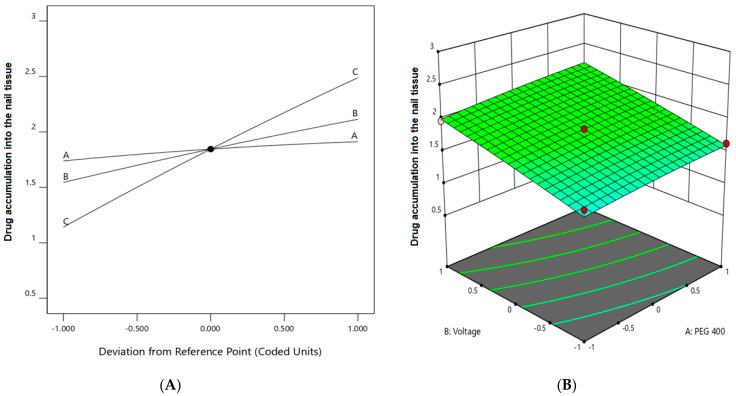
Effect of the independent variables on drug accumulation into the nail tissue (**A**) perturbation plot and (**B**) 3D surface plot.

**Figure 6:**
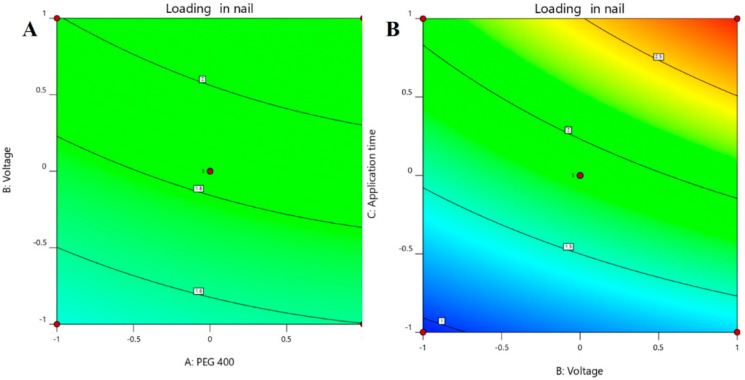
Contour plot response showing the effect of independent variables on the permeation of drug (**A**) voltage versus PEG 400 and (**B**) application time versus voltage.

The multilinear equations relating to coded factors are employed in carrying out predictions about the response for given levels of individual factors. The coded equation is beneficial for assessing the comparative effect of the factors by relating the coefficient values. The positive coefficients of all the three model terms (i.e., A, B, and C) in Equation (2) represent an increase in drug accumulation into the nail tissue with the increase in PEG 400 content in the formulation, and also with increasing application time and applied voltage. The influence of each independent variable is indicated in Table 5 where all the model terms are found to be significantly affecting the drug accumulation (*p* < 0.05); however, the influence of model term C (i.e., application time) shows the highest (0.6738) coefficient value in the Equation (2) when compared to the others (i.e., 0.0865 and 0.2846 for A and B, respectively), which indicated the maximum response on drug accumulation into the nail tissue with changes of model term C. On the other hand, the model term B shows a higher effect when compared to model term A, which is indicated by their respective coefficient values in Equation (2). A similar influence of the interaction of independent variables on the drug accumulation into the nail tissue was depicted in the perturbation plot (Figure 5A) and the 3D surface plot (Figure 5B). It is well understood from the figures that the effect of all the three independent variables coincides with the previous explanation on the quadratic equation. In addition, the comparison of predicted and actual values of this analysis has been presented in Table 2 and Figure 3B, where the closeness of the actual and predicted values are noticeable. On the other hand, from the contour plot response, it is evident that the changes in drug accumulation into the nail tissue are much higher with the rise in applied voltage when compared to the increase in PEG content in the formulation (Figure 6A). Contrarily, the application time has shown a higher impact on the drug accumulation into the nail tissue when compared to the applied voltage (Figure 6B). Our findings on increase in drug accumulation into the nail tissue with the increase in PEG 400 content in the formulation have also been demonstrated in the reported literature [46]. Furthermore, the application of increased voltage during the iontophoresis process has been established to have a direct relationship with drug accumulation into the nail tissue [55]. Similarly, the duration of application of the formulation at the site is also having a direct relationship with the drug accumulation into the nail tissue [37].

#### 3.3.3. Optimization and Validation

From the optimization process, with the selected desired criteria on the permeability of drug and drug accumulation into the nail tissue, using Design-Expert software, it was suggested that the formulation with 30% PEG 400 could provide the terbinafine permeation of 53.82 µg/cm^2^ and drug accumulation of 2.86 µg/mg in a nail when the applied voltage is 12 V, which is in well below the human tolerable voltage (30 V) [56], with an application time of 6 h. Overall the results suggests that the maximum effective values for permeation and drug accumulation into the nail tissue could be achieved using PEG 400 (30% *w*/*w*), voltage (12 V), and duration (6 h). Thus, the composition of the optimized formulation has been fabricated with 30% (*v*/*v*) PEG 400 for the next step of the experiment.

**Figure 7:**
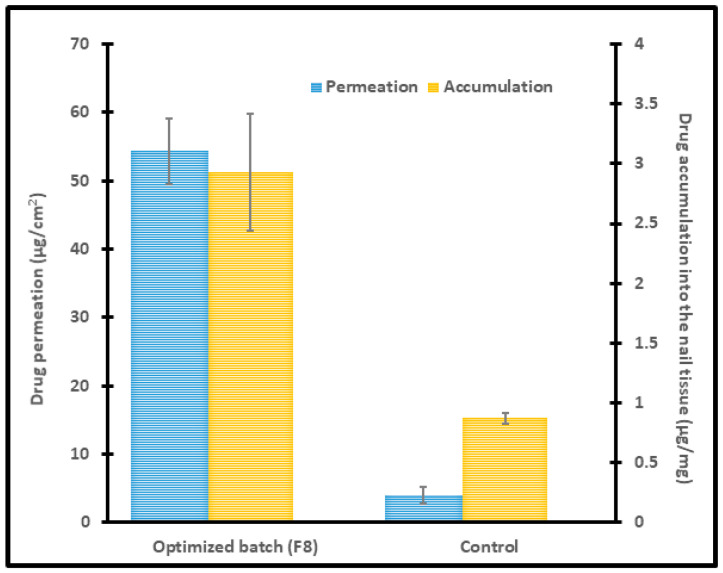
Permeation and drug accumulation into the nail tissue by optimized batch (F8) using iontophoresis (12 V for 6 h) and control (0 V for 6 h). The data represents mean ± SD (*n* = 6).

The obtained experimental data for optimized batch (F8) on the drug permeability and drug accumulation into the nail tissue were presented in Figure 7, which are in close agreement with the predicted values with a difference of 1% and 2.3%, respectively. A significant enhancement in permeation and drug accumulation was noticed by the optimized batch, signifying the potential of constant voltage iontophoresis in transungual drug delivery. Moreover, it has been demonstrated that the drug permeation and accumulation into the nail tissue did not differ among the diseased and healthy nails [57]. Hence it is presumed that the data observed here could be translated into onychomycotic nails.

### 3.4. Characterization of Optimized formulation

#### 3.4.1. FTIR

**Figure 8:**
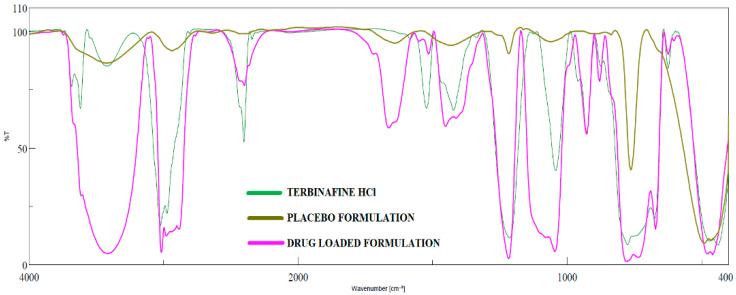
FTIR spectra of terbinafine, placebo formulation, and optimized formulation (F8).

Possible chemical interaction between drug and excipients in pharmaceutical products is usually assessed during formulation development by measuring the FTIR spectra of drug, placebo, and formulation. In general, the drug–excipient compatibility studies have been used to determine whether the additives in the product interact with the drug that may lead to drug decomposition, which in turn affects product stability [58]. FTIR spectra of terbinafine, placebo, and formulation (F8) are shown in Figure 8. Pure terbinafine displays C=C stretching bands at 1523.49 cm^−1^, aromatic C≡C stretching bands at 2402.87 cm^−1^, aromatic C–H stretching bands at 3023.84 cm^−1^, and C−N bands at 1423.21 cm^−1^ [59]. Identical peaks were shown in the IR spectra of formulation signifies no drug–excipient interaction. It revealed C≡C stretching bands at 1515.78 cm^−1^, aromatic C–H stretching bands at 3016.12 cm^−1^, aromatic C≡C stretching bands at 2399.01 cm^−1^, and C–N bands at 1454.06 cm^−1^. The predominant peaks for terbinafine and F8 were confirmed with the theoretical estimation corresponding to the functional groups. However, the masjor peaks of terbinafine are not observed in placebo formulation.

#### 3.4.2. DSC

**Figure 9:**
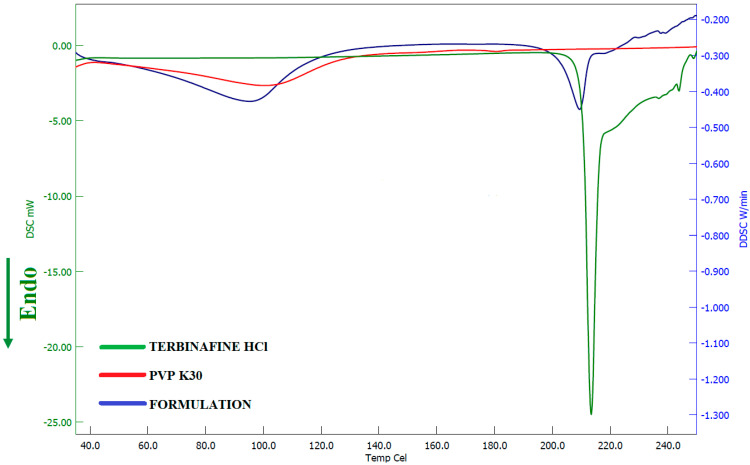
Differential scanning calorimetric patterns of terbinafine, polyvinylpyrrolidone K 30, and optimized batch (F8).

The thermal behavior of pure drug, PVP K30, and the formulation was evaluated using DSC and the corresponding thermograms were exhibited in Figure 9. The thermogram of pure terbinafine showed a characteristic endothermic peak at 213.5 °C related to its melting point and decomposition [60]. On the other hand, the DSC spectra for PVP K30 showed an endothermic peak at 100.01 °C. The Thermogram of formulation exhibited a characteristic peak of PVP K30 while the intensity of the drug characteristic peak was reduced and shifted to a slightly lower temperature 209.4 °C confirming its amorphization. Overall, the data here revealed that the pure drug and excipients are compatible in the formulation.

#### 3.4.3. SEM

**Figure 10:**
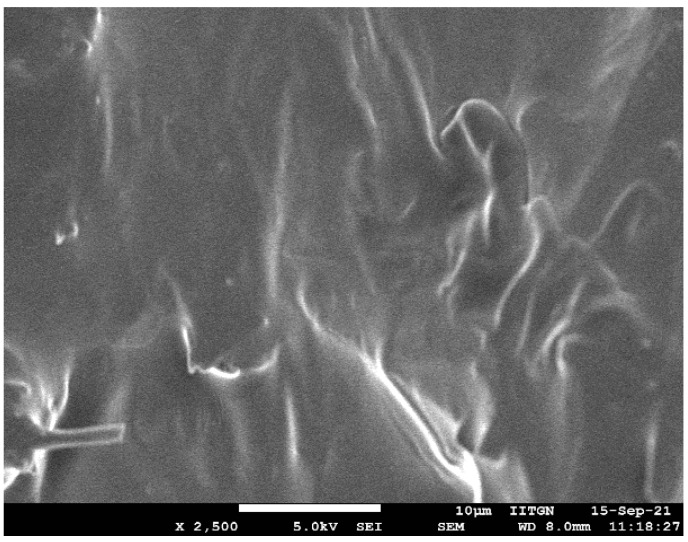
Representative scanning electron microscopy image of optimized formulation (F8).

SEM micrograph of formulation F8 is depicted in Figure 10. Visual observation of the image indicates complete miscibility of terbinafine with the formulation components and no visible presence of drug crystals. The film-like structure displayed in the image could be probably because of the polyvinylpyrrolidone K 30 present in the formulation. The non-uniformity in the surface could be likely due to the aggregation of formulation ingredients, and may not essentially represent the hydrated product as described in the literature [61].

**Figure 11:**
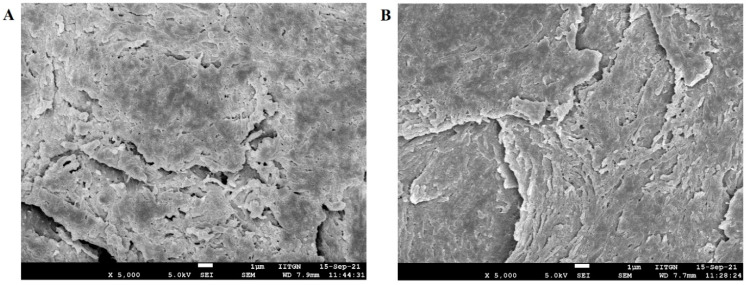
Scanning electron microscopy images of (**A**) treated nail plate (iontophoresis, 12 V for 6 h using formulation F8) and (**B**) control nail (passive, 0 V for 6 h using formulation F8).

SEM micrographs of human nail plates treated with iontophoresis (12 V for 6 h using formulation F8) and control (passive, 0 V for 6 h using formulation F8) were captured to compare the topography, texture, and morphology of the dorsal surface. It is evident from Figure 11 that the structural morphology of both treated and control nail plates was similar. In both cases, the surface cells seem to be close, did not show much disruption (though few apertures were visible), and have not altered the overall integrity of the nail structure. Indeed, the results here signify that the low voltage iontophoresis did not alter the structure of the nail, which is also in agreement with the general observation in constant current iontophoresis [62].

### 3.5. Drug Release from Nail

**Figure 12:**
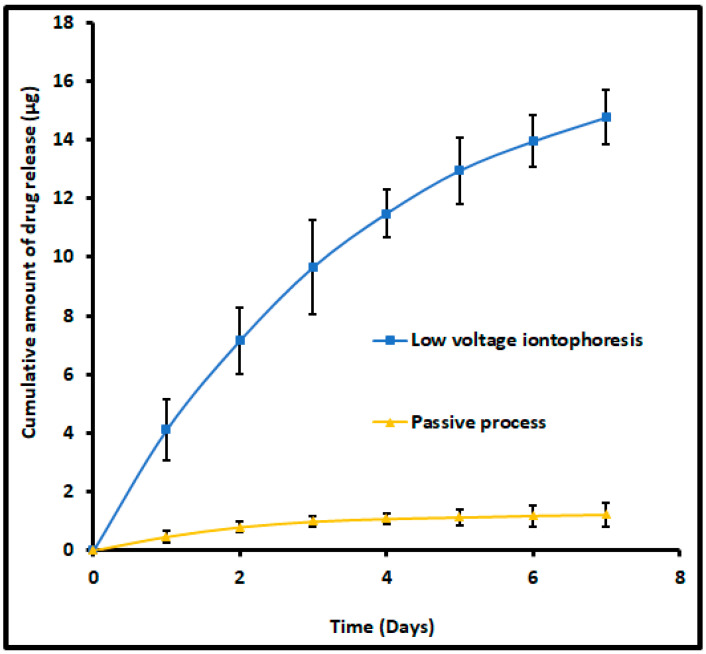
Comparison of the amount of terbinafine release into the receiver from drug-accumulated nails by low voltage iontophoresis (F8) and passive process (control). The drug accumulation into the nail tissue was done with an optimized batch (F8) using iontophoresis (12 V for 6 h) and a passive process (0 V for 6 h). The data represents mean ± SD (*n* = 6).

The release of terbinafine from the drug encapsulated nail plates is of prime importance. It is a well-known fact that the retention of drugs in the nail plate and its subsequent release is considered a crucial factor, particularly for topical nail products. This is mainly because the drug depot in the nail plate ultimately releases into the nail bed, where the organism resides. Therefore, the ex vivo release experiments were conducted for nail plates comprising terbinafine by iontophoresis (12 V for 6 h) or passive (0 V for 6 h). The release profiles of terbinafine from nails accumulated by iontophoresis and passive process were shown in Figure 12. Two distinct release profiles were observed for nails accumulated with terbinafine by iontophoresis and passive process. A greater amount of drug release (*p* < 0.0005) was noticed in nails accumulated by iontophoresis as compared to nail accumulated by the passive process in the current experimental conditions. In addition, the nails accumulated with terbinafine by iontophoresis, the release rate was high during the first day demonstrating a maximum amount released as 4.12 ± 1.04 µg, and decreased steadily further with duration and released ~0.8 µg at day seven (Figure 12). The rationale for this high drug release rate in the initial period could be directly attributed to the existence of a relatively higher amount of free terbinafine in the nail plate, which eventually diffused out [37]. Followed by the free drug release, the drug molecules embedded in the nail or bound to the keratin occurred. The total amount released at the end of seven days was 14.76 ± 0.92 µg, which represents ~40% of the total drug accumulated in nail plates (based on the average value of drug accumulated in nail plates found in batch F8 during iontophoresis). These results signify that a higher payload of the drug could be released from the depot formed in the nail matrix into the nail bed by iontophoresis application. However, the cumulative drug release from the nail accumulated by the passive process was considerably low (1.23 ± 0.41 µg in 7 days), in addition to the slow release rate observed throughout the study period. This less amount of drug release could be directly related to the total drug accumulation in passive delivery, which itself is considerably low, and is consistent with earlier studies [38]. In general, ex vivo release results disclosed that there was a pronounced release of the drug across the nail plates when the drug was accumulated by iontophoresis and was statistically significant (*p* < 0.005) when compared to the release of the drug across the nails when the drug was accumulated by passive delivery. Thus, the release occurring across the nails from the drug accumulated by iontophoresis is likely to provide continuous release of drug over at least a week and could serve to bathe the nail bed in clinical situations that could reduce the frequency of topical therapy, though need to be investigated in vivo.

### 3.6. Antifungal Activity

**Figure 13:**
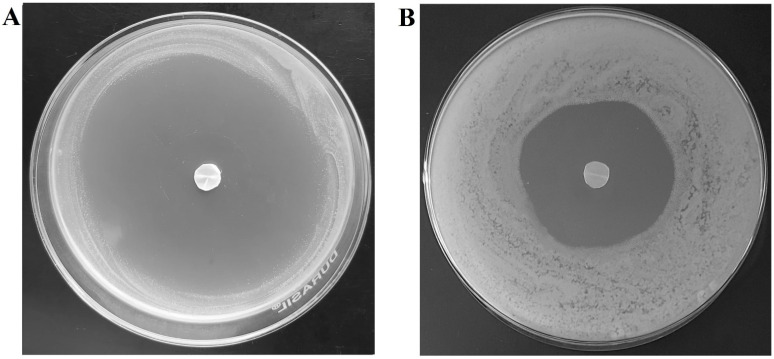
Photographic images showing antifungal efficacy by the drug-accumulated nail plates at day 4 by (**A**) iontophoresis and (**B**) passive process. The drug accumulation into the nail tissue was done with optimized formulation (F8) using iontophoresis (12 V for 6 h) and passive process (0 V for 6 h).

The most critical factor in the antifungal activity of a topical nail formulation relies on the amount of drug passing through the nail as well as the drug loading inside the nail plate that could release and exhibit therapeutic activity (pharmacodynamic effect) [63]. Therefore, antifungal activity of terbinafine-accumulated nail plates (by iontophoresis and passive process) were tested against *Trichophyton mentagrophyte* and the results were compared based on the zone of inhibition surrounding the nail plate at day 4 (Figure 13). The method used here is based on the release of drugs from the terbinafine-accumulated nails and then diffuses into the agar and inhibits the microbial growth, which implies an antifungal effect. As shown in Figure 13, the zone of inhibition is remarkable (which is 200% greater; *p* < 0.001) in the case of iontophoretic accumulated nail plates (77.9 ± 2.6 mm) as compared to the passive process (38.4 ± 1.7 mm). A possible explanation for such enhanced fungicidal activity of iontophoretic accumulated nail could be potentially due to the higher drug accumulation that happens due to iontophoresis (2.93 ± 0.49 µg/mg) than the passive counterpart (0.87 ± 0.05 µg/mg). This data also substantiates the release data, wherein the drug release was significantly higher in iontophoretic accumulated nails than passive. On the other hand, the low inhibition zone observed in nails accumulated by the passive process suggests less antifungal activity. Overall, the data here signifies that the drug depot in the nail will ultimately release and shows the antifungal effect, which could significantly improve the success rate of topical nail therapy in onychomycosis.

### 3.7. Stability

**Figure 14:**
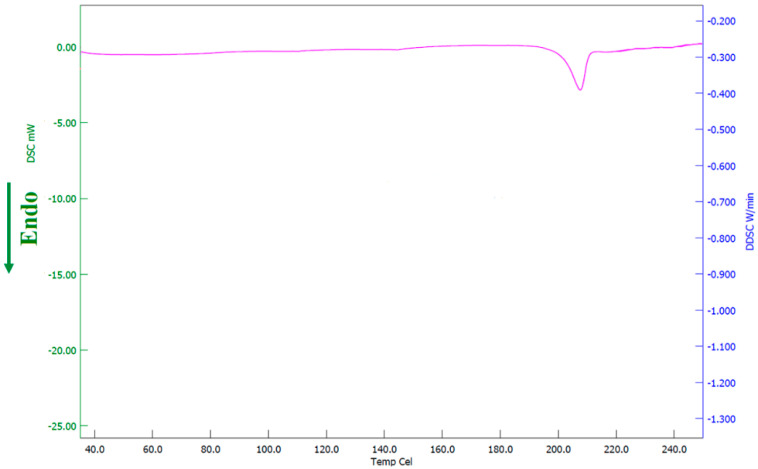
Differential scanning calorimetric patterns of optimized batch (F8) after stability testing.

Stability data indicate that the formulation used in the F8 batch was stable during the study period. The product remains homogeneous without any lump formation or sedimentation. Moreover, the formulation demonstrated no significant variation in the drug loading (96.34 ± 1.90%), pH (3.59 ± 0.22), or viscosity (65.2 ± 3.61 cP at 30 rpm) during storage. Similarly, the thermogram of formulation (Figure 14) was comparable to the spectra observed with formulation before the stability period (Figure 9), suggesting no variation in the amorphous state of the drug. Overall, the data signifies that the formulation had good physical and chemical stability for 3 months.

## 4. Conclusions

This study successfully evaluated the prospect of the constant voltage iontophoresis technique for the transungual delivery of terbinafine. Box–Behnken statistical design was carried out to optimize the formulation development and terbinafine permeation across the nail plate and drug accumulation into the nail tissue of the selected formulation. High throughput screening of various chemicals has identified PEG 400 as the potential nail permeation enhancer for terbinafine and was included in the formulation. Preliminary studies suggested that the PEG 400, voltage, and application time can influence both transungual permeation and drug accumulation into the nail tissue and were selected as independent variables. Optimization data revealed that all three independent variables significantly influence the terbinafine delivery and the optimized batch F8 (with 30% PEG 400, an applied voltage of 12 V, and application time 6 h) demonstrated noticeable improvement in permeation and drug accumulation into the nail tissue. It is evident from the drug release and antifungal studies that the drug depot formed in the nail plates by iontophoresis will release in due course and exert the desired fungicidal activity. Thus, the results signify that the drug-accumulated by low voltage iontophoresis is advantageous as it could eventually reduce the frequency of application and sustain therapy in clinical settings. Indeed, one can easily vary either the voltage or application time to accomplish the required dose of the drug to be delivered to the nail plate. In summary, the obtained data demonstrated here would provide a new approach towards the efficient treatment of onychomycosis, though needs to be established in clinical studies. Furthermore, the constant voltage iontophoresis approach could be easily applied in clinical practice by designing a suitable patch type, a self-wearable iontophoretic device with a 12 V battery, which will ultimately improve the pharmacotherapy against onychomycosis

## Figures and Tables

**Table 1:** Composition of preliminary batches (T1–T6) of gels.

Ingredients	Batch Code					
	T1	T2	T3	T4	T5	T6
Terbinafine HCl (% *w*/*w*)	4	4	4	4	4	4
Ethanol (% *w*/*w*)	20	20	20	20	20	20
Tween 80 (% *w*/*w*)	5	5	5	5	5	5
Polyethylene glycol 200 (% *w*/*w*)	15	-	30	-	30	-
Polyethylene glycol 400 (% *w*/*w*)	-	15	-	30	-	30
Propylene glycol (% *w*/*w*)	10	10	10	10	10	10
Polyvinylpyrrolidone K 30 (% *w*/*w*)	10	10	10	10	20	20
Water as required to (% *w*/*w*)	100	100	100	100	100	100

**Table 2:** Physicochemical characteristics of preliminary batches (T1–T6).

Parameters	Batch Code					
	T1	T2	T3	T4	T5	T6
pH	3.39 ± 0.21	3.48 ± 0.37	3.26 ± 0.18	3.51 ± 0.24	3.22 ± 0.15	3.44 ± 0.26
Loading efficacy (%)	97.41 ± 3.67	96.57 ± 3.74	97.05 ± 3.28	98.22 ± 2.62	96.88 ± 3.11	96.37 ± 3.34
Drug loading (%)	3.90 ± 0.15	3.86 ± 0.14	3.88 ± 0.13	3.93 ± 0.10	3.88 ± 0.12	3.85 ± 0.13
Viscosity (cP) at 30 rpm	62.5 ± 2.65	65.1 ± 1.98	65.9 ± 2.21	68.2 ± 1.50	73.3 ± 2.75	75.6 ± 2.31
Cumulative amount permeated in 6 h (µg/cm^2^)	1.08 ± 0.26	2.67 ± 0.74	1.83 ± 0.45	3.97 ± 1.18	1.69 ± 0.42	3.80 ± 1.24
Drug accumulation into the nail tissue in 6 h (µg/mg)	0.22 ± 0.02	0.42 ± 0.05	0.33 ± 0.04	0.87 ± 0.05	0.29 ± 0.03	0.69 ± 0.07

**Table 3:** Representation of levels of independent variables based formulation details (F1–F17) with data of actual and predicted responses in Box–Behnken design.

Batch Code	Values of Independent Variables			Actual Responses		Predicted Responses	
	A	B	C	R1	R2	R1	R2
	(% *w*/*w*)	(V)	(h)	(µg/cm^2^)	(µg/mg)	(µg/cm^2^)	(µg/mg)
F1	0	0	0	32.63 ± 4.76	1.85 ± 0.23	32.63	1.85
F2	1	1	0	38.54 ± 4.84	2.10 ± 0.30	39.11	2.19
F3	0	0	0	32.63 ± 4.76	1.85 ± 0.23	32.63	1.85
F4	0	0	0	32.63 ± 4.76	1.85 ± 0.23	32.63	1.85
F5	−1	0	1	42.38 ± 4.55	2.36 ± 0.39	42.68	2.41
F6	0	−1	1	31.26 ± 2.77	2.04 ± 0.33	31.54	2.09
F7	0	−1	−1	11.64 ± 3.00	0.87 ±0. 24	12.17	0.94
F8	0	1	1	54.35 ± 4.76	2.93 ± 0.49	53.82	2.86
F9	−1	−1	0	22.78 ± 3.66	1.55 ± 0.27	22.20	1.45
F10	1	0	1	45.17 ± 5.93	2.56 ± 0.30	45.12	2.53
F11	1	0	−1	18.23 ± 3.64	1.27 ± 0.25	17.94	1.23
F12	−1	0	−1	16.71 ± 2.14	0.99 ± 0.19	16.75	1.01
F13	0	1	−1	20.37 ± 4.23	1.36 ± 0.28	20.09	1.31
F14	−1	1	0	36.03 ± 5.93	1.97 ± 0.24	36.27	1.99
F15	0	0	0	32.63 ± 4.76	1.85 ± 0.23	32.63	1.85
F16	0	0	0	32.63 ± 4.76	1.85 ± 0.23	32.63	1.85
F17	1	−1	0	23.21 ± 4.25	1.63 ± 0.22	22.98	1.60
Independent variable		Low (−1)		Medium (0)		Upper (+1)	
A = PEG 400 (% *w*/*w*)		20		30		40	
B = Voltage (V)		6		9		12	
C = Application time (h)		2		4		6	
Dependent variables: R1 = Permeation (µg/cm^2^) R2 = Accumulation into the nail tissue (µg/mg)							

**Table 4:** The ANOVA data generated through software from the quadratic model on trans-nail drug permeation.

Source	Permeation		
	Sum of Squares	F-Value	*p*-Value
Model	1952.29	911.96	<0.0001
A-PEG 400	6.57	27.63	0.0012
B-Voltage	456.15	1917.71	<0.0001
C-Application time	1410.16	5928.47	<0.0001
AB	1.08	4.54	0.0705
AC	0.3964	1.67	0.2377
BC	51.57	216.82	<0.0001
A^2^	1.69	7.12	0.0320
B^2^	14.45	60.77	0.0001
C^2^	7.91	33.26	0.0007
Residual	1.67		
Lack of Fit	1.67		
Pure Error	0.0000		
Cor Total	1953.96		

**Table 5:** ANOVA data generated by the software for quadratic model on drug accumulation into the nail tissue.

Source	Accumulation into the Nail Tissue		
	Sum of Squares	F-Value	*p*-Value
Model	4.39	87.16	<0.0001
A-PEG 400	0.0599	10.70	0.0137
B-Voltage	0.6479	115.76	<0.0001
C-Application time	3.63	648.86	<0.0001
AB	0.0007	0.1275	0.7316
AC	0.0021	0.3792	0.5575
BC	0.0413	7.39	0.0299
A^2^	0.0017	0.2958	0.6034
B^2^	0.0010	0.1874	0.6781
C^2^	0.0038	0.6739	0.4388
Residual	0.0392		
Lack of Fit	0.0392		
Pure Error	0.0000		
Cor Total	4.43		

## Data Availability

The data presented in this study are contained within the article.
